# Clinical Readiness for Practice of Nursing Students: A Concept Analysis

**DOI:** 10.3390/ijerph21121610

**Published:** 2024-11-30

**Authors:** Kennedy Diema Konlan, Dulamsuren Damiran, Tae Wha Lee

**Affiliations:** 1 Department of Public Health Nursing, School of Nursing and Midwifery, University of Health and Allied Sciences, Ho P.O. Box 31, Ghana; dkkonlan@uhas.edu.gh; 2 College of Nursing, Brain Korea 21 FOUR Project, Mo-Im Kim Institute, Yonsei University, Seoul 03722, Republic of Korea; twlee5@yuhs.ac; 3 School of Nursing, International University of Ulaanbaatar, P.O. Box 658 Namyangjuu Street, 25th khorrlol, Bayangol District, Ulaanbaatar 2106448, Mongolia

**Keywords:** readiness, clinical practice, nursing, nursing students

## Abstract

Introduction: The concept of clinical readiness for practice among nursing students is yet to be analyzed, and there is a lack of empirical evidence on its usage among academics and clinicians. Methods: This concept analysis is anchored on a systematic literature review that adhered to the PRISMA guidelines and incorporated the eight iterative steps of Walker and Avant’s concept analysis method. This concept analysis method involved: (1) choosing a concept; (2) determining the objectives of the analysis; (3) identifying usages of the concept; (4) determining the defining attributes; (5) identifying a model case; (6) identifying other cases, including borderline, contrary, and related cases; (7) identifying antecedents and consequences; and (8) defining empirical references. The integrative thematic data synthesis method was adopted. Results: The concept of nursing students’ clinical readiness for practice is said to have four interrelated attributes. These attributes included (1) professional skills, (2) communication skills, (3) self-management skills, and (4) self-confidence. The two antecedents for nursing students’ clinical readiness to practice are (1) personal factors, including demographic characteristics, prior healthcare experience, income, and emotional intelligence; and (2) educational factors, including the clinical learning environment, clinical internship program, learning resource, and learning strategy. The consequence of clinical readiness for the practice of nursing students includes obtaining practice skills that can lead to more personal and job-related satisfactory outcomes. Conclusions: clinical readiness for practice in nursing encompasses the acquisition and integration of professional knowledge, skills, effective communication abilities, and self-management capabilities and the application of these competencies with confidence toward the provision of high-quality care to patients. Clinical Relevance: Understanding the components of clinical readiness is crucial for nursing educators, preceptors, and healthcare institutions to ensure that nursing students are adequately prepared for the challenges they will face in clinical practice. By recognizing the importance of professional knowledge, skills, communication, and self-management in clinical readiness, educators and training institutions can tailor their curricula, programs, and support systems to better prepare nursing students for the demands of real-world healthcare settings. This focus on clinical readiness ultimately delivers safe, effective, and compassionate patient care.

## 1. Background

There is a gap between the clinical environment and the theory taught in the classroom, which can harm professional practice and result in a scarcity of health professionals [1]. Nurses’ professional practice requires a careful and efficient combination of clinical and theoretical knowledge, usually obtained from classroom teachings. When nurses are equipped with these two skills, they have a superior likelihood of showing improved practice and efficiency and can meet the real demands of the job; hence, they may be deemed ready for practice [2]. Therefore, clinical readiness for practice is a cornerstone for the intricacies associated with the nursing profession [2]. However, recent studies in Ohio, the United States; Victoria, Australia; and Shiraz, Iran, discovered that graduating nurses lacked clinical skills, with only a small percentage possessing entry-level abilities and readiness for clinical practice [3].

Nursing schools are expected to produce nurses who, despite their lack of experience, are clinically ready to practice to meet the increasing healthcare demands of an aging population and to mitigate the projected nursing shortage [4]. The ability of graduate nurses to transition smoothly into clinical practice is dependent on their ability to acculturate into the social context of their work, especially during training or immediately after [2,5], developing effective emotional intelligence [6] and having considerable stress-free access to jobs and transitioning [7,8]. However, many nursing graduates who pass the state licensing exam may be unprepared to work in complex clinical fields [2,3]. The perception that graduate nurses are unprepared for clinical practice, referred to as the theory–practice gap, is influenced by a variety of factors, including the divide between educational institutions and the clinical setting, the quality of training opportunities and support in undergraduate clinical placements, and socialization into the nursing profession [2,3,9]. Consequently, nurses’ transitioning to clinical practice or professional practice may be challenging because of inadequate clinical readiness to practice [10]. As a result, some newly qualified nurses may suffer emotional fatigue and burnout leading to disillusionment or lack of motivation to provide professional services [11,12].

The concept of readiness for practice received some description in recent years and hence is devoid of some ambiguity [13,14]. However, these descriptions mainly focused on new nurses [13] and evidence-based practice readiness [14]. Consequently, some scholars used these concepts in their works [13,14]. Yet, there is increasing ambiguity and uncertainty in the use and understanding of the concept of clinical readiness for the practice of student nurses among academicians and scholars. This may be associated with the fact that clinical readiness for practice has not been analyzed, yet there is a lack of empirical evidence of its usage among academics and clinicians alike. Consequently, conducting a concept analysis to properly delineate the concept of clinical readiness for practice for nursing students is currently warranted, as it will lead to clarity on the meaning and usage and provide a basis for making empirical references for its operationalization. This concept analysis aims to provide a clear and concise definition of the concept of clinical readiness for practice for nursing students.

## 2. Methods

This concept analysis is anchored on a systematic literature review that adhered to the PRISMA guidelines for conducting reviews.

### 2.1. Design

This concept analysis used Walker and Avant’s [15] eight-iterative steps to identify and give appropriate delineation to clinical readiness for the practice of nursing students. Concept analysis was described as a strategy that allows researchers to examine the attributes and characteristics of a concept [15]. This process allows for the purging of unambiguous meaning from any new or existing concept whose usage is unclear and generally appears to be difficult to operationalize or measure [15]. The Walker and Avant concept analysis method is a robust, efficient, and simple method that is relatively popular, as it provides the basis for creating cases (e.g., model, contrary related), identifying antecedents and consequences, as well as clearly stating the defining attributes of the concept [15]. The reasons for choosing this concept analysis method were its simplicity and straightforwardness, as well as the use of cases to expose further the concept under consideration. In addition, the Walker and Avant concept analysis method allows for focusing on available literature obtained through systematic literature review and consequently allows the concept to be delineated in available empirical literature. The Walker and Avants’ [15] concept analysis method usually involves eight iterative steps that include: (1) choosing a concept; (2) determining the objectives of the analysis; (3) identifying usages of the concept; (4) determining the defining attributes; (5) identifying a model case; (6) identifying other cases, including related cases; (7) identifying antecedents and consequences; and (8) defining empirical references.

### 2.2. Literature Search and Screening

The search keywords were anchored on the population, concept, and context (PCC) framework. This was because our goal was to retrieve all eligible studies using a scoping search methodology. The keywords were used as specific terms, synonyms, or medical subject headings (MeSH) where it was applicable and were linked using the appropriate Boolean operators, wildcards, and truncation. Using the suitable Boolean operators, the search was performed using the words/phrases; “readiness”, “for clinical practice”, “clinical readiness”, “nursing” and “graduate nurses”, and “trainees”, and “students”. The search was restricted to the years 1980–2023 and in the English language. The search from the three databases included the Cumulative Index for Nursing and Allied Health Literature (CINAHL) (1313), PubMed (4407), and Embase (153) databases, and one paper was retrieved from the reference list of the selected papers, resulting in 5874 titles. The retrieved studies were transported to an EndNote (version 20) referencing software, and 1420 duplicate titles were identified through electronic and manual searches.

**Figure 1:**
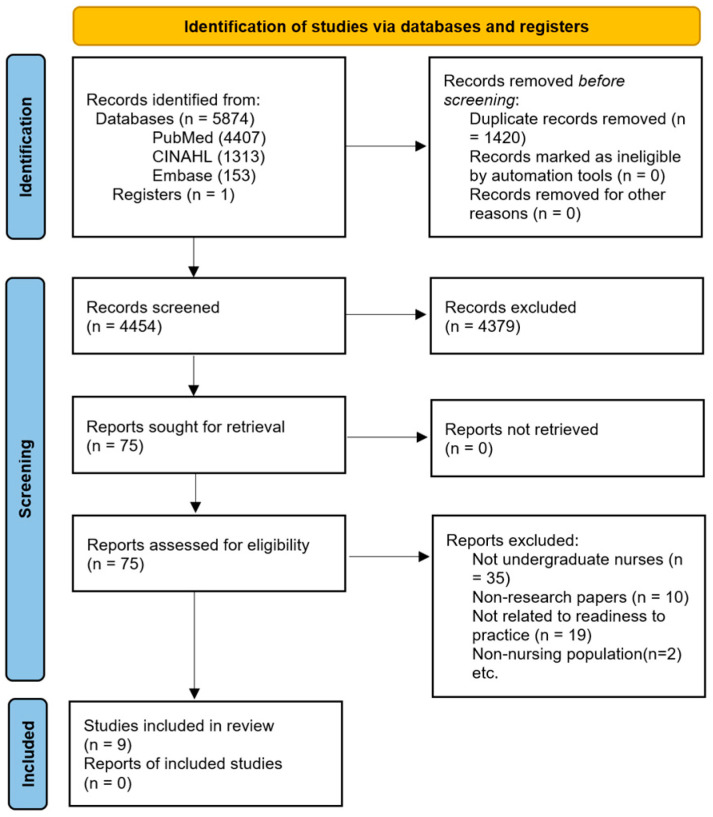
Study selection flow chart.

Eventually, 4454 titles were screened by title and abstract and 75 were selected for full text review. In the title and abstract screen stage, 1421 studies were excluded because they did not focus on (1) nursing students, (2) clinical readiness for practice, or (3) were not scientific research papers. A study was included for further evaluation if the title or abstract stated any of the aforementioned concepts or their related synonym. The 75 studies that progressed to the full-text screening stage were meticulously screened against similar parameters. At this stage, a study was qualified if it met all of these criteria, including (1) involved undergraduate students in the population, (2) was a scientific empirical study, clearly using scientific methodology, and (3) described readiness for practice. Some of the studies did not meet the criteria because they did not involve undergraduate nurses (10), non-research papers (10), and they were not related to readiness to practice (19) or the nursing population (2). As a result, nine studies were selected for this study (Figure 1).

### 2.3. Data Analysis and Synthesis

The integrative data synthesis method was adopted for this concept analysis. As a result, the findings from the selected studies were first extracted using a data extraction matrix individually, compared, collated, and agreed upon by all the researchers (KDK, DD). The extracted data, depending on the various steps of the Walker and Avant concept analysis methods, were then transformed into specific qualitative descriptions according to the principles enshrined in thematic data analysis [16,17]. Therefore, a thematic data analysis method was adopted and synthesized for the critical defining attributes, the antecedents, and the consequences. To achieve this, the Akinyode (2018) [18] thematic data analysis method was adhered to. This method of thematic data analysis involved six steps that included (1) familiarization with the data, (2) generating the initial codes, (3) searching for themes, (4) reviewing and defining the themes, (5) organizing the themes into coherent structures (such as attribute, antecedents, and consequences), and (6) producing the analysis report as a concept [18]. As a result, there was an independent line-by-line coding of the individual data extracted from the selected papers by forming free codes that were merged into subthemes and subsequently the main themes and corresponded to the various aspects of the steps of Walker and Avant’s concept analysis method. The cases were then developed based on the researchers’ experiences working as nursing trainers both in clinical and classroom settings [15].

## 3. Results

The results of this study are presented in line with the eight-step iterative concept analysis method that Walker and Avant (2005) proposed [15].

### 3.1. Study Characteristics

The studies were conducted in three different countries, including (1) Asia, India [1,3], (2) America [19,20,21], and (3) Australia [22,23,24,25] (Table 1). The study designs used were systematic review and metanalysis [22], mixed methods study [25], pre-test post-test survey [24], qualitative and descriptive [23], and cross-sectional [1,3,20,21].

### 3.2. Walker and Avant’s Concept Analysis Methods

This current concept analysis method adhered to Walker and Avant’s (2005) method [15]. This process allows for giving unambiguous meaning to any new or existing concept. These are usually constructed in usage, and also when the concepts used are ambiguous and generally appear to be difficult to operationalize or measure. The concept of clinical readiness for the practice of nursing students has been used to refer to diverse and multiple constructs and is hence associated with a level of misunderstanding and sometimes misapplication among researchers. Therefore, clarifying the defining attributes, the antecedents, and the consequences of clinical readiness for the practice of nursing students through concept analysis is warranted.

#### 3.2.1. Select a Concept

The concept of clinical readiness for the practice of nursing students is not well defined. The concept is used to refer to the immediate capacity to manage clinical scenarios expected by more experienced professionals in a particular discipline [26,27]. This concept has been used variedly and mostly differently. For example, some researchers highlighted a lack of confidence while speaking with physicians, patients, and family members as perceived defects in clinical readiness [9,28]. Additionally, diverse and multiple concepts have been used as synonyms for clinical readiness for practice, with the critical challenge that these constructs are different [29]. Others describe it as transition shock, or acute stress associated with negotiating new professional relationships, tasks, duties, and work–life balance, as well as adjusting to the physical demands of clinical practice, which has been linked to new nurses’ integration into the workforce [29].

Recognizing that clinical readiness for practice is an important component of nursing and a crucial determinant of access to and utilization of health care services and patient outcomes, conducting a concept analysis will be useful to promote nursing practice research and education. While readiness to practice, especially among newly qualified graduate nurses, received some form of concept analysis, thereby producing some uniformity in usage, clinical readiness to practice among student nurses has not been specifically addressed, hence the diverse ambiguity. Therefore, the goal of this concept analysis was to give clear delineation and uniformity in the understanding and usage of the concept of clinical readiness of nursing students by defining its attributes, antecedents, and consequences and illustrating using appropriate cases.

#### 3.2.2. Determine the Aims of the Analysis

Due to the ambiguity and multiplicity in the usage of the concept of clinical readiness for the practice of nursing, conducting a concept analysis was imperative. The primary goal of this concept analysis was to describe and explain the use and meaning of the concept of clinical readiness to practice and to identify the main attributes or characteristics, its antecedents and consequences, and develop appropriate cases.

#### 3.2.3. Determine All of the Concept’s Applications

Even though several scholars define clinical readiness for practice, several meanings and interpretations persist among scholars. For example, Candella and Bowles (2008) identified clinical readiness as managing a large patient caseload and caring for critically ill patients [30]. Clinical readiness in nursing is defined as the ability to perform a task, procedure, technique, or activity [31]. Clinical practice readiness refers to the application of industry-related skills such as teamwork, time management, communication skills, social skills, and emotional intelligence [5]. Additionally, clinical practice readiness was described as having a reasonably good level of basic nursing skills as a graduating nurse [3].

From an analysis of the aforementioned, clinical readiness for the practice of nursing students still has inherent ambiguity in use. Clinical readiness for practice is used in the following ways according to the literature: the attainment of a reasonably good level of basic nursing skills; the ability to perform a task, procedure, technique, or activity; having sufficient confidence when communicating with physicians, patients, and family members; demonstrating critical knowledge; and integrating sufficiently into the industry (hospitals/communities) with improved related skills.

#### 3.2.4. Determine the Defining Attributes

**Figure 2:**
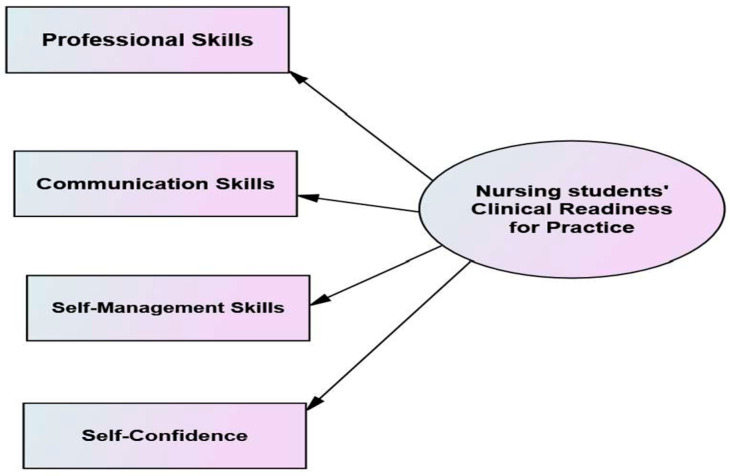
The defining attributes of nursing students’ clinical readiness for practice.

Walker and Avant defined attributes as “qualities or characteristics that are frequently associated with a concept, and providing additional insight into its meaning” [15]. Additionally, defining attributes describe those characteristics that must be present for the concept to be identified and distinguished from related concepts [15]. In this study, the attributes were identified through the integration of related factors through the qualitative thematic data analysis technique. The concept of nursing students’ clinical readiness for practice is said to have three interrelated attributes. These attributes included (1) professional skills, (2) communication skills, (3) self-management skills, and (4) self-confidence. The first three are already identified as attributes associated with readiness for practice [13], while self-confidence is seen as an additional critical component for clinical readiness for practice among student nurses. Additionally, the attribute of graduate students’ clinical readiness to practice identified a level of self-confidence (behavior) in clinical nursing service, expressed in a new nurse’s behavior in terms of how well the knowledge, skills, communication, and self-management are implemented in clinical settings. Figure 2 shows the defining attributes of nursing students’ clinical readiness for practice.

#### 3.2.5. Identifying Antecedents and Consequences

Antecedents and consequences are critical in delineating the associated factors of a concept under consideration.

##### Antecedents

**Figure 3:**
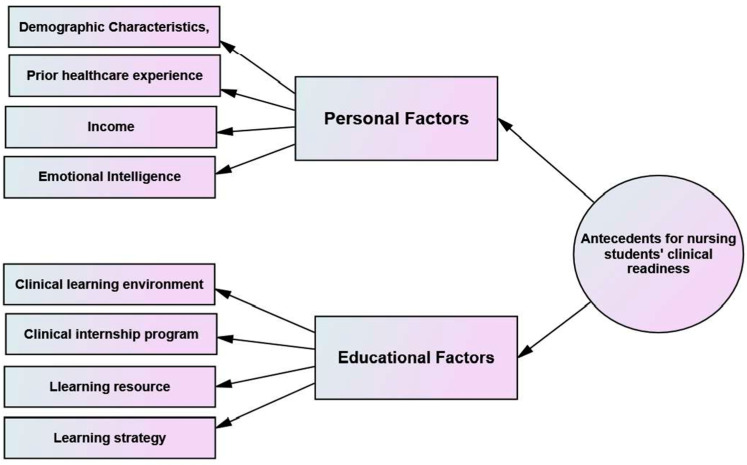
Antecedents of nursing students’ clinical readiness for practice.

Walker and Avant describe antecedents as the foundational elements of a concept [15]. In this study, the antecedents for nursing students’ clinical readiness for practice were identified by an integrative thematic analysis of the findings of the included studies. The two antecedents for nursing students’ clinical readiness for practice are (1) personal factors, including demographic characteristics, prior healthcare experience, income, and emotional intelligence [1,19,22], and (2) educational factors, including the clinical learning environment [1,3,19], clinical internship program, learning recourse, and learning strategy [3,19,22,25]. Figure 3 shows the antecedents of nursing students’ clinical readiness for practice.

##### Consequences

**Figure 4:**
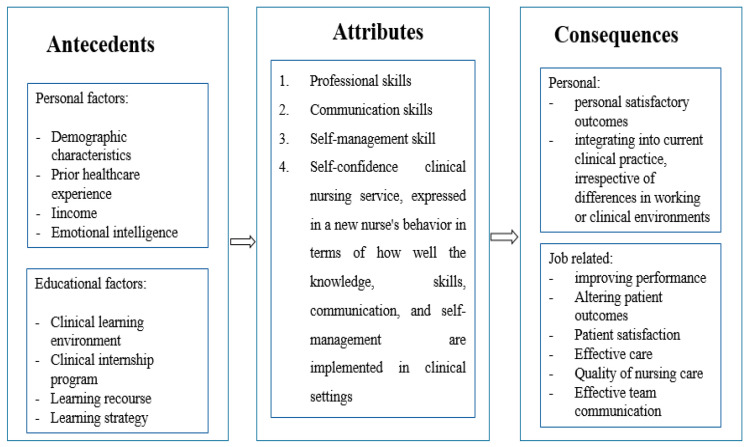
Concept of clinical readiness for practice among nursing students: antecedents, attributes, and consequences.

Consequences refer to those factors that are a result of the concept or an outcome of a concept. Consequently, it refers to the positive/negative product of a particular concept under consideration. Evidence-based approaches to producing more confident clinical students through increased practice preparedness result in registered nurses who can give safe and effective patient care independently [19]. New graduates with clinical readiness practice skills could have more personal and job-related satisfactory outcomes [3,22,24,25]. Personal outcomes will be expressed by integrating into current clinical practice, irrespective of differences in working or clinical environments [21,23]. Additionally, the job-related outcomes that were identified as the consequence of student nurses’ clinical readiness to practice were improving performance and altering patient outcomes, patient satisfaction, effective care, quality of nursing care, and effective team communication [3,22,25] (Figure 4).

#### 3.2.6. Identify Cases

##### Model Case

The model case refers to the scenario that presents all the different and varied perspectives on the concept. The module case, as described by Walker and Avants, showed that if this is not the concept, what else can be? [15]. This is because, usually, the model case presents all the defining attributes of the concept, the antecedents, and the consequences. In this study, the model case presents a case deduced from many years of work experience by the authors. The model case is used to exemplify all the critical defining attributes of the concept [15]. In this current study, the model cases expressed all the defining attributes of the concept.

Madam JA is a 31-year-old newly employed nurse (antecedent–demographic younger age) at the Tamale Teaching Hospital. She received 4 years of training in general nursing from the University for Development Studies (antecedent–educational factors). Before her enrolment in the university, she had 5 years of working experience as a clinical ward aid with six months of training experience (antecedent–personal factors). Madam JA believes that she received adequate and appropriate training that increased her knowledge, skill, and responsibility and ensured that the activities that she engages in are with the utmost ability and grounded in science (self-confidence). She was not disturbed when she was posted to work in the facility (no transition shock). Since her posting to the ward, where she sometimes works as a shift in charge, there has been tremendous improvement in the quality of care rendered to patients in that unit because she and other nurses have taken it as a responsibility to improve the interpersonal relationship with patients. (attributes–professional skills). She worked with two other nurses in the Unitarch consortium to determine the net influence of the regimented diabetes insulin administration on the long-term control of blood sugar among elderly patients (attributes–professional skills). She also ensures that basic clinical bulletins related to specific disease conditions in the ward are distributed via a WhatsApp platform as well as the ward’s notice boards (attributes–communicative skills). Many of her colleagues agreed that her presence tremendously improved the nature of clinical practice. Madam JA believes that she is contributing in her capacity as a nurse to the development of the hospital and the country at large (consequences–personal outcome). Two months ago, due to the net improvement in patient outcomes in the facility, she was given the “best nurse shift in charge award” by her colleagues in the unit. In her citation, the nurses indicate she spearheaded improvement in performance outcomes for the preceding year, increased patient satisfaction, ensured quality nursing care, and promoted effective team communication (consequences–job-related outcomes).

In this model case, it can be seen that the scenario presents all the antecedents, defining attributes, and consequences of the concept of nursing students’ clinical readiness for practice.

#### 3.2.7. Identify Additional Cases

Walker and Avant (2005) argued that adding cases was critical for further showing the defining characteristics of a concept under consideration [15]. In this study, we added two more additional cases that showed the borderline case and the contrary case.

##### Borderline Case

The borderline case presents a scenario where only some aspects of the concept are shown, as not all the defining attributes can readily be deduced. In the current study, this borderline case was also identified through long periods of practice by the authors. The borderline case is usually used to clarify the concept under consideration. The borderline case may not exactly fit into an example of the case under consideration, but it is said to exhibit some of the defining attributes of the concept.

Madam AR is 24 years old, recently completed college with a diploma in general nursing, and had no prior experience working in the hospital. She had all her clinical placements scheduled in a primary health care center located in the remote resource-limited rural setting where she lived. Her school was generally deprived and lacked many clinical resources including some faculty (lacks some critical antecedents—personal and educational factors). She was recently posted to a teaching hospital and this generated much anxiety and fear in her (transition shock). She thinks that she will have challenges coping with the system and working with sophisticated gadgets in the hospital (lacks self-confidence). Upon assumption of duty, most of her superiors are worried about her sluggishness to work, lack of confidence, and inability to have full control of a ward (lacks professional and self-management skills). However, Madam AR is considerate of patients’ concerns, communicates efficiently according to cultural norms, and is empathetic (has good communication skills). Consequently, Madam AR has limited job satisfaction, has not made any other consideration to further her education, and is having challenges in integrating into the health care system. Recently, she was chosen to undergo in-service training to improve her clinical skills, yet patients and her superiors still complain about her inability to perform nursing procedures with confidence, lack of effective teamwork ability, and poor quality of services (consequence, lack of personal and job-related outcome).

In this borderline case, Madam AR exhibits some of the antecedents as some demographic and education characteristics. Additionally, other defining attributes were not exhibited (self-confidence, professional and self-management skills) while others were exhibited (transition shock, communicative skills). As a result, not all of the consequences of the concept were then observed (personal and job-related outcomes).

##### Contrary Case

This refers to the lack of the defining attributes of the concept under consideration [15]. This is very important to state because it adds to the internal dialogue used to examine the defining attributes [15]. The contrary case is usually used to exemplify what is ‘not the concept’. In this case, the contrary case does not seem to portray any of the defining attributes of nursing students’ clinical readiness for practice.

Madam DF is a 55-year-old nurse assistant trained through the 1980s Ghana government’s special program of apprenticeship to enroll health aids in government hospitals. She has never been to a nurses training college and has no form of advanced training (lacks antecedents–personal and educational). She was posted to a secondary health facility and has since worked there. Even though she can perform some basic nursing procedures, she is particularly challenged to manage a ward alone. She sometimes feels frustrated, especially regarding patients who complain of chronic pain. Madam DF also has a challenge communicating with patients and other healthcare professionals in the hospital (lacks the defining attributes). In a recent case, she had an altercation with the charge nurse and was served a disciplinary measure; this frustrated her such that she refused to come to work for three weeks. She has no plans for personal or professional development and is currently preparing for her retirement. Her job output according to patients has also been poor (lack of consequence).

In this case, it can be seen that Madam DF lacks the antecedents, the defining attributes, and the consequences of the concept under consideration. This then makes the case an example of a contrary case.

#### 3.2.8. Defining Empirical Referents

In their work, Walker and Avant (2005) described empirical referents as ways to measure the concepts in the real world. It also refers to the empirical ways to quantify or identify the concept or to distinguish the concept from related ones [15].

The measurement of nursing students’ clinical readiness for practice mostly focuses on levels of self-confidence, including skills for subscale. Several studies of clinical readiness for practice are used to classify levels, seen as a whole, into categories, such as 5-point Likert scale-type involving skills. Other studies assign scores to levels of specified components of clinical readiness for practice to obtain a numerical index usually ranging from 0 to 100. Empirical referents are vital because they support the concept’s validity by providing ways to measure the concept. The nurse’s skills are important for clinical practice readiness. Thus, it is useful in future new nurses’ self-confidence to include consequences in quality evaluation. It will be useful to research the field to identify clinical practice readiness for nurses and improve education strategies based on the evidence.

## 4. Discussion

This current concept analysis evaluated an important concept regarding the eventual output related to the training of nursing students by conducting a systematic review adhering to the Prisma guidelines and synthesizing the defining attributes, antecedents, and consequences using the integrative thematic data analysis method. The eventual results ensured that critical models were constructed to practically outline and give meaning to those characteristics. The entire concept of “nursing students clinical readiness for the practice”, defining attributes (personal factors-demographic characteristics, prior health experience; and education factors-clinical learning environment, clinical internship program, learning course, and teaching strategy), antecedents (self-confidence, professional skills, communication skills, and self-management skills), consequences (personal outcome-satisfied with their job, able to integrate into current practice, and job-related outcome—improving performance and outcome, patient satisfaction, effective care, quality of nursing care, and effective team communication) and the build cases was carried out noting the very importance of the concept in the formulation of nursing theory, nursing practice, and nursing education.

One important characteristic of nursing students’ clinical readiness for practice was self-assurance, which included professional, communication skills, and self-management skills. Consequently, these outcomes resulted in personal outcomes as well as job-related outcomes, both of which are critical in nursing service. It is therefore imperative that nurse scientists develop interventions that can promote the self-assurance of nursing students. Additionally, another grey area for conducting concept analysis is the concept of “self-assurance”. This will provide a critical basis for formulating theories that can promote the clinical practice of students and promote their eventual integration into service [32]. This is important because most researchers, clinicians, and practitioners appear to confuse self-assurance with self-confidence, hence making concept analysis imperative [33,34]. Appropriately analyzing these concepts can promote bridging the gap between clinical practice and nursing science [34].

This current concept analysis was conducted by noting that the concept of “readiness for practice” [13] had already been analyzed, yet there were some specific discrepancies in how the concept can be used in clinical practice. Therefore, this detailed definition of nursing students’ clinical readiness to practice deviates from previous concept analyses that did not include all the diverse perspectives and only focused on the broader concept of readiness to practice. Additionally, previous definitions showed that clinical readiness to practice is described as the immediate capacity to manage clinical scenarios that a more experienced nurse would expect on rare occasions [26,27]. This definition is further limited because of its ability to show all the critical defining attributes, antecedents, and consequences. The current review further broadens the concept, as it shows the defining attributes of the concept to include self-confidence, the absence of transition shock, professional skills, communication skills, and self-management skills.

### 4.1. The Theoretical Implications for the Concept Analysis

Concept analysis methods must form the basis for the formulation and sometimes the clinical application of concepts. This is because a theory is built on diversely formulated concepts [15]. A theorist introduces the reader to the critical defining attributes using theoretical definitions, which are usually abstract and may not be measurable, and are usually a conglomeration of diverse but related concepts. The theoretical definition, as well as possible application of the concept of nursing students’ clinical readiness for practice, refers to the attainment of self-confidence, which includes professional skills, communication skills, and self-management skills, with the defined goal of achieving self-reliance, self-assurance, scientific perspective, and the provision of acceptable organized care to patients and communities. Understanding and formulating theories regarding nursing students’ clinical readiness for practice is important [11,35] because of the increasing numbers of undergraduate students, overcrowding in clinical space during training, decreasing clinical placement hours, and a decreasing motivation of clinical preceptors’ influence on workplace readiness [36,37].

As nursing theory is still developing, very few focused on the gateway to clinical practice and the level of readiness always associated with student nurses’ practice. Given that there is a depth of information regarding the concept of nursing students’ clinical readiness to practice, this current concept analysis in the context of developing countries highlights the need to begin to consider the concept among students as the “honeymoon” for most graduating students tends to become shorter. This concept analysis may be a preliminary point to consider developing a nursing theory for understanding, explaining, and providing critical underpinnings for the provision of healthcare services to patients and communities. Developing a nursing theory (middle range) will propel a better appreciation of the challenges that newly qualified nurses face and allow for putting in pragmatic measures that will be critical in mitigating the related challenges. This is particularly important in that caring for multiple patients involves a high level of dexterity in a complex environment that warrants a steep learning curve and can only be attained through building the student’s nurse confidence, clinical skills, and overall competence within a facilitative environment.

### 4.2. Strengths and Limitations

This concept analysis method adopted the robust Walker and Avant’s methods that have been used in the nursing discipline to explain, describe, or appropriately identify the critical attributes of unclear or ambiguous terms [38]. The use of this concept analysis method allowed for showing diverse and multiple perspectives. This was achieved because of the construction of cases. The development of the cases exemplified the concept and contextualized it in real-life situations. By using this concept analysis method for nursing students’ clinical readiness for practice, one developed an appreciation for the complexity of the concept through the use of a systematic literature review process that added to the reliability of the incorporated scientific process. However, inherent in this review process are some critical limitations that ought to be acknowledged. One important limitation was the identification of only papers that were published in the English language, limiting the possible generalization of the findings in other languages. Additionally, this concept analysis approach adopted the scoping review methodology for screening and selecting included studies, as the PCC framework was adopted for literature search. This allowed for the inclusion of different studies with varied study designs.

## 5. Conclusions

This concept analysis identified the antecedents, defining attributes, and consequences of the nursing students’ clinical readiness for practice. The most important characteristic is self-assurance, which includes professional skills, communication skills, and self-management skills. These outcomes resulted in personal outcomes as well as job-related outcomes, both of which are critical in nursing service. This conceptualization of nursing students’ clinical readiness to practice defines what it means to be a clinically practice-ready new nursing graduate. Finally, the proposed definition of the integrated terms of nursing students’ clinical readiness for practice is “acquiring professional nursing knowledge, skills, communication skills, self-management skills, and the reasonable application of these skills with the self-confidence to deliver quality care.” after providing this succinct definition for nursing students’ clinical readiness for practice, it is hoped that nursing researchers will begin to consider varied methodologies for developing and nursing theory to describe, explain, or promote the concept.

## Figures and Tables

**Table 1:** Study characteristics and key findings.

Author	Purpose	Population	Method	Country	Attributes	Antecedents	Consequences
Davies et al., 2021 [22]	Determined extended immersive ward-based simulation improves the preparedness of baccalaureate nursing students.	N = 12 Paper	Systematic reviews and meta-analysis	Australia	Extended immersive ward-based simulation	Reduced anxiety and improves self-confidence	Improve the sustainability of the nursing workforce and reduce early career burnout.
Reid-Searl et al., 2021 [25]	Enhanced undergraduate nursing students’ work readiness and confidence to care for children.	N = 22 Undergraduate nursing students	A mixed methods study	Australia	Improvements in pediatric nursing skills in clinical learning,	Clinical reasoning, and clinical confidence	More prepared with clinical reasoning and confidence skills in the clinical practice.
Sharma et al., 2020 [3]	Assessed the clinical practice readiness of nursing graduates.	N = 173 Final-year nursing students	Cross-sectional study	India	Age (younger), Clinical longer duration, School type	Clinical nursing competence	More prepared in clinical practice.
Munroe and Loerzel, 2016 [20]	Evaluated the level of genomic literacy among undergraduate nursing students and assessed attitudes, readiness, and comfort toward using knowledge in clinical practice.	N = 120 Nursing students	Cross-sectional study	USA	Nursing curricula provided more focused on genomic education	Knowledge of clinical practice	Comfortable and proficient in providing care for patients with genomic conditions.
Ericson and Zimmerman, 2020 [19]	Explored the impact of CCE on ADN students’ and nurse preceptors’ views of practice preparedness.	N = 57 Student -23; Preceptor-34	Retrospective study	USA	Professional identity, ability to manage many patients, advanced skills	Competence, and professional identity	Confident with the clinical environment.
Kaur et al., 2020 [1]	Investigated senior nursing students’ opinions of readiness to practice and the impediments that prevent them from doing so.	N = 176 Outgoing graduate nurses	Descriptive cross-sectional survey	India	Ability to identify and appropriately document clinical errors.	Better remuneration	In total, 50% were not prepared for clinical practice due to barriers.
Porter et al., 2013 [24]	Measured final-year nursing students’ confidence in clinical skill performance and confidence in high-acuity clinical placement.	N = 483 Final-year nursing students	Pretest/posttest survey design	Austalia	High-acuity clinical placement	Confidence	Clinical skill was increased.
Musallam and Flinders, 2021 [21]	Explored the impacts of COVID-19 on the students’ perceptions of readiness for practice and their preparation for the NCLEX exam.	N = 26 senior BSN students	Cross-sectional descriptive design	USA	Clinical experiences, Ability to perform clinical service	Level of comfort in performing nursing roles	Impact on career opportunities and plans.
Kerr et al., 2020 [23]	Explored the perspectives of final-year, undergraduate student nurses regarding the introduction of a directed self-guidance strategy and the self-directed learning laboratory.	N = 12 Final-year baccalaureate nursing students	Qualitative, and descriptive method	Australia	Directed self guidance and SDL lab	Enhanced confidence during clinical placements	Improve accessibility and contemporaneous equipment and layout might enhance realism.

## Data Availability

There are no specific clinical resources declared as all relevant information sources are included in the reference list.
